# New Membrane-Forming Aromatic Co-Poly(amide-imide)s: Influence of the Chemical Structure on the Morphological, Thermal and Transport Properties

**DOI:** 10.3390/membranes12010091

**Published:** 2022-01-14

**Authors:** Svetlana V. Kononova, Danila A. Kuznetsov, Galina N. Gubanova, Elena V. Kruchinina, Anatoly Ya. Volkov, Milana E. Vylegzhanina, Elena N. Vlasova, Boris Z. Volchek

**Affiliations:** Institute of Macromolecular Compounds, Russian Academy of Science, Bolshoy pr. 31, 199004 Saint-Petersburg, Russia; 79216366850@yandex.ru (D.A.K.); gubanovagn@yandex.ru (G.N.G.); evkruchinina@mail.ru (E.V.K.); volanya@bk.ru (A.Y.V.); v.e.milana@gmail.com (M.E.V.); evl021960@gmail.com (E.N.V.); spectra@imc.macro.ru (B.Z.V.)

**Keywords:** poly(amide-imide), copolymers, dense and phase inversion membranes, structure and morphology, pervaporation

## Abstract

Polymer film membranes are used to solve specific separation problems that dictate structural requirements. Structural and morphological parameters of film membranes based on glassy polyheteroarylenes can be controlled in the process of preparation from solutions that opens up prospects for obtaining structured membranes required for targeted separation. In the case of aromatic poly(amide-imide)s, the possibility of controlling film formation and structure virtually has not been studied. In the present work, a series of homologous co-poly(amide-imide)s differing in the number of repeating units with carboxyl-substituted aromatic fragments was synthesized by polycondensation. Comparative analysis of the processes of formation of membranes with different morphologies based on these polymers under equal conditions was performed. New information was obtained about the influence of the amounts of carboxyl groups and the residual solvent on structural properties of asymmetric membranes. The influence of these factors on transport properties of dense membranes under pervaporation conditions was studied. It was demonstrated that in the case of carboxyl-containing poly(amide-imide)s, the domains formed during film preparation had a significant effect on membrane properties.

## 1. Introduction 

Aromatic poly(amide-imide)s (PAIs) have proven to be promising membrane-forming polymers [1,2,3,4,5,6,7,8,9,10,11,12,13,14]. Due to their ability to form defect-free dense films with good mechanical characteristics (for example, E ≈ 3.79 GPa [14]), they are used in manufacturing membranes of various morphologies. 

A special advantage of poly(amide-imide) polymers is their solubility in amide solvents and other media; thus, poly(amide-imide) films can be prepared both by free evaporation of a solvent from the casting solution and by the phase inversion process. 

The demand for highly efficient membranes has stimulated development of phase inversion films based on these polymers [2,3,4,5,6,7,8,9,10,11,12,13,14]. They include not only diffusion asymmetric membranes for pervaporation separation of liquids, but also ultra- and nanoporous membranes that serve as substrates in composite diffusion membranes. A large number of research works are devoted to improving structure of these membranes and to optimizing processes of formation of porous films under phase inversion conditions.

However, modern experts in membrane separation are faced with several pressing problems, such as the problem of selective separation of gases and liquids with high permeability or the matter of separation and concentration of nano-scale viruses from various aqueous media. Thus, it has become necessary to design membranes containing active functional groups on the surface. Although the problems of gas/liquid separation and the issue of virus separation seem completely different, these two types of processes impose similar requirements on membrane surface modification. In the first case, an ultrathin polymer separation layer should be formed on the denser surface of an asymmetric membrane; besides, this layer should be chemically bound to the dense membrane. In the second case, it is necessary to add a nano-porous skin layer with a certain number of functional groups that exert some influence on the filtration process.

Much attention has been given to selection and optimization of preparation conditions for porous membranes of complex morphologies based on aromatic poly(amide-imide)s. General regularities of the formation of phase inversion membranes have been investigated, and detailed studies of the structure of these membranes have been performed. In our previous studies, it has been shown that introduction of diamine fragments of different polarities into a polymer in the process of polycondensation made it possible to directly control diffusion properties of the resulting membranes [11,14]. At the same time, it was possible to vary polymer chain rigidity and to introduce the necessary functional groups.

The main distinguishing feature of the syntheses carried out in our work was introducing a monomer containing an imide fragment into polycondensation reaction [15]. This technique enabled us to avoid cyclization of polyamic acids that leads to formation of polyimides. Several poly(amide-imide)s were obtained by one-stage low-temperature polycondensation of 4-chloroformyl-(N-p-chloroformylphenyl) phthalimide with various diamines, such as diaminodiphenyl ether (PAI-1), sulfur-containing diamines, and other similar compounds [14].

The experience of working with poly(amide-imide)s containing fluorine-substituted aromatic fragments showed that the introduction into the molecule of groups that do not fundamentally change the degree of flexibility of the polymer chain, but lead to partial hydrophobization of the polymer, is reflected in the morphology of the corresponding phase-inversion membranes [16,17]. In other words, the widespread statement that asymmetric porous structures that are practically identical in morphology are formed from polymers of the same class with similar chemical structures under the same conditions of the wet molding process using hard or moderately hard precipitators is not accurate. Our earlier research has shown that under the same conditions, poly(amide-imide)s containing different diamine components in macrochains gave structurally different asymmetric microporous membranes characterized by similar morphologies in the skin layer area [8,14,16,17].

Using PAI-1 as an example, we have developed preparation methods for membranes with the necessary structural and morphological elements (membrane structure control). The effect of compositions of the precipitation bath and the casting solution, as well as the influence of post-processing, on membrane morphology has been revealed [14,17]. The obtained membranes had one common characteristic feature: the presence of macropores that cross the entire inner part of the film (cross section) and become tapering near the upper surface. The density of the skin layer and pore walls depended on precipitator parameters. When a mixture of precipitants was substituted for precipitation baths, no major changes in film morphologies were observed. However, in this case, a wide area with smaller pores appeared near the skin layer; these pores were elongated towards the upper surface. This observation turned out to be useful in obtaining membranes that are stable under the conditions of baro membrane processes [17,18].

The mechanism of formation of phase-inversion membranes in the form of gradient porous films of glassy polymers is widely discussed in the literature [2,8,17,18,19,20]. A series of our previous research works [8,17,21,22] was motivated by the lack of literature data on possible morphological differences between membranes of various polymer homologs of the class of aromatic poly(amide-imide)s, and by the absence of information about optimization of preparation conditions for poly(amide-imide) reverse-phase membranes.

Special attention was given to the study of supramolecular structure of poly(amide-imide)s in the skin layers of phase inversion membranes, to investigation of pore formation and development of visualization methods [21,22].

In the work of Kononova et al. [8], poly(amide-imide)s with diphenyl ether fragments in polymer chains were compared with those containing 3,5-N,N-disubstituted diaminobenzoic acid fragments (PAI-2). Mechanical and thermo-physical properties of nonporous dense membranes based on PAI-1 and PAI-2 differ significantly. It was shown that nonporous PAI-2 films formed under the same conditions as PAI-1 films have significantly different morphological features.

**Figure 1:**
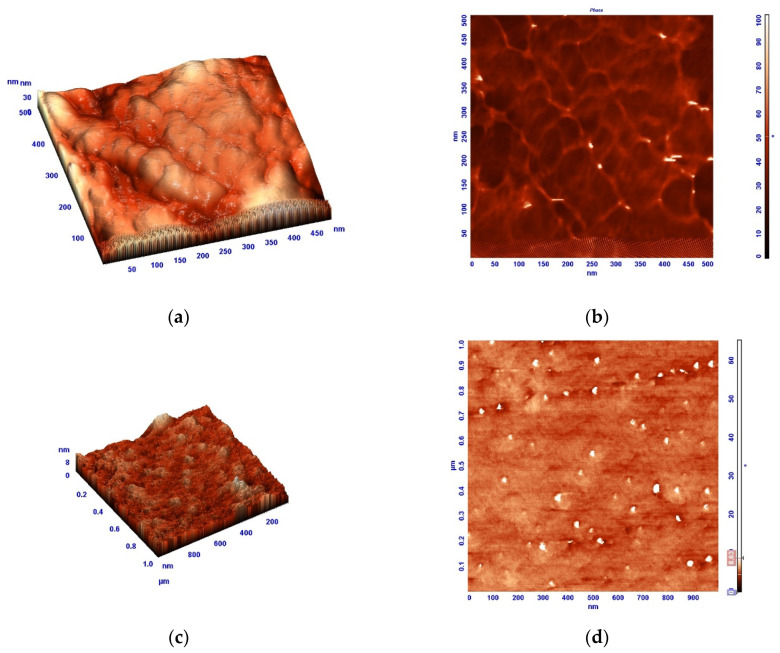
AFM images of upper layers of nonporous dense PAI-1 (**a**,**b**) and PAI-2 (**c**,**d**) films ((**a**,**c**)—3D images, (**b**,**d**)—images obtained in the phase contrast mode) [8].

AFM images (3D image and the image taken in the phase contrast mode) of the upper surface (the polymer–air interface) of a nonporous PAI-2 film are shown in Figure 1. The surface layer of the film contains polymer domains; however, they are smaller than those on the PAI-1 surface. Carboxyl groups present in the PAI-2 sample exhibit high affinity for the evaporating solvent, which causes changes in polymer chain conformation; in particular, “solvent exit craters” are formed whose presence is revealed by the phase-contrast SEM image.

For PAI-2, water is a softer precipitant than for PAI-1. This leads to the formation of asymmetric porous structure with thicker pore walls and skin layer. However, the influence of precipitant characteristics (“softness”) on the morphology of the film formed under the wet spinning conditions has not yet been studied. This question is especially interesting because studies of formation of PAI-1 membranes in various precipitators have shown that the results can be unexpected due to many factors affecting the system.

In the present study, new copolymers containing fragments of 4,4′-diaminodiphenyl ether and 3,5-diaminobenzoic acid in different ratios were synthesized; the influence of carboxyl-containing fragments on structural, morphological, and transport properties of continuous nonporous and phase-inversion PAI membranes was investigated.

## 2. Experimental Section

### 2.1. Materials

#### 2.1.1. Reagents

4,4′-diaminodiphenyl ether and 3,5-diaminobenzoic acid were of reagent grade, supplied by Sigma-Aldrich (St. Louis, MO, USA) and were used without further purification. 4-Chloroformyl (N-p-chloroformylphenyl) phthalimide was dried for 24 h, 4,4′-diaminodiphenyl ether and 3,5-diaminobenzoic acid were dried for 48 h; then the reagents were dried under vacuum. N-Methyl-2-pyrrolidone (NMP) supplied by Sigma-Aldrich (St. Louis, MO, USA) was used without further purification. In the experiments, distilled water (pervaporation study) and deionized water (contact angles measurements) were used which is obtained on a laboratory installation immediately before the study.

#### 2.1.2. Polymer Synthesis

**Figure 2:**
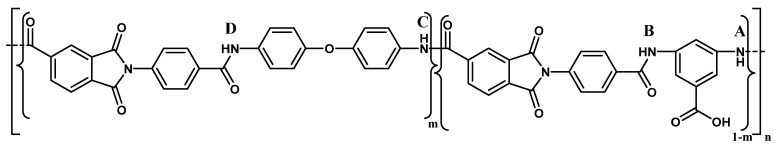
Structural formulas of PAI-1 (m = 1), PAI-2 (m = 0) and copolymers coPAI-1 (m = 0.7), coPAI-2 (m = 0.5), coPAI-3 (m = 0.3); letters are labels assigned to protons.

Poly(amide-imide)s (PAIs, see the general formula in Figure 2) were synthesized from 4-chloroformyl (N-p-chloroformylphenyl) phthalimide and 4,4′-diaminodiphenyl ether (PAI-1) or 3,5-diaminobenzoic acid (PAI-2) by low-temperature polycondensation in solution according to the slightly modified method described in [15]. The reaction was carried out in N-methyl-2-pyrrolidone (N-MP); the mixture was cooled down to −15 °C for 1 h, then left to stand at room temperature (for not less than 10 h) until a viscous solution was formed. The synthesized polymer was isolated from the reaction mixture and purified by repeated precipitation in water and alcohol baths. After removal of the solvent, the polymer in the form of a powder was studied by FTIR and NMR spectroscopy (Bruker spectrophotometer equipped with a Pike attachment with a ZnSe working element (Billerica, MA, USA); NMR spectrometer AVANCE II-500 WB from Bruker (Billerica, MA, USA)).

**coPAI-1.** To a solution of 1.47 g (7.35 mmol) of diaminodiphenyl ether (DADPhE) and 0.4788 g (3.15 mmol) of 3,5-diaminobenzoic acid (DABA) in 30 mL N-MP cooled to 0–5 °C were added 3.764 g (10.815 mmol) of 4-chloroformyl-(N-p-chloroformylphenyl)phthalimide. The mixture was left to stand until it heated up to room temperature, and was kept at room temperature for 1 h. Then 14 mL of N-MP were added, and the mixture was stirred for 2 h.

**coPAI-2**. The coPAI-2 was synthesized by the same method as coPAI-1 using 1.2 g (6.00 mmol) DADPhE, 0.912 g (6.00 mmol) 3,5-diaminobenzoic acid, 4.3013 g (12.36 mmol) 4-chloroformyl-(N-p-chloroformylphenyl)phthalimide, 1.6 mL of oxypropylene and overall 48 mL N-MP.

**coPAI-3**. The coPAI-3 was synthesized by the same method as coPAI-1 using 0.6 g (3.00 mmol) DADPhE, 1.064 g (7.00 mmol) DABA, 3.5844 g (10.30 mmol) 4-chloroformyl-(N-p-chloroformylphenyl)phthalimide, 1.4 mL oxypropylene. The total amount of used N-MP was 40 mL.

#### 2.1.3. Polymers Characterization

**The reduced viscosity** η_red_ of a 0.5 wt.% solution of each synthesized PAI was determined in N-MP at 20 °C for characterization solutions using for film formation. 0.05 g of dry polymer (for each PAI under investigation) was dissolved in 10 mL of N-MP. Outflow times of N-MP and 0.5 wt.% PAI solutions were measured by use of an Ostwald viscometer.

The reduced viscosity was estimated from the outflow time of N-MP and 0.5 wt.% solutions of PAI in N-MP by the equation

(1) $$\eta_{\mathrm{red}}=\left(\frac{t_{s}}{t_{N-MP}\;}-1\right)/c$$

where *t_s_* is the solution outflow time; *t_N-MP_*, *N-MП*—outflow time; *c*, solution concentration (g/dL).

The reduced viscosity values (0.5 wt.% solutions in N-MP) of the synthesized polymers PAI-1, PAI-2, coPAI-1, coPAI-2, coPAI-3 were equal to 2.20, 2.05, 2.69, 2.38, and 2.00 dL/g, respectively.

**Figure 3:**
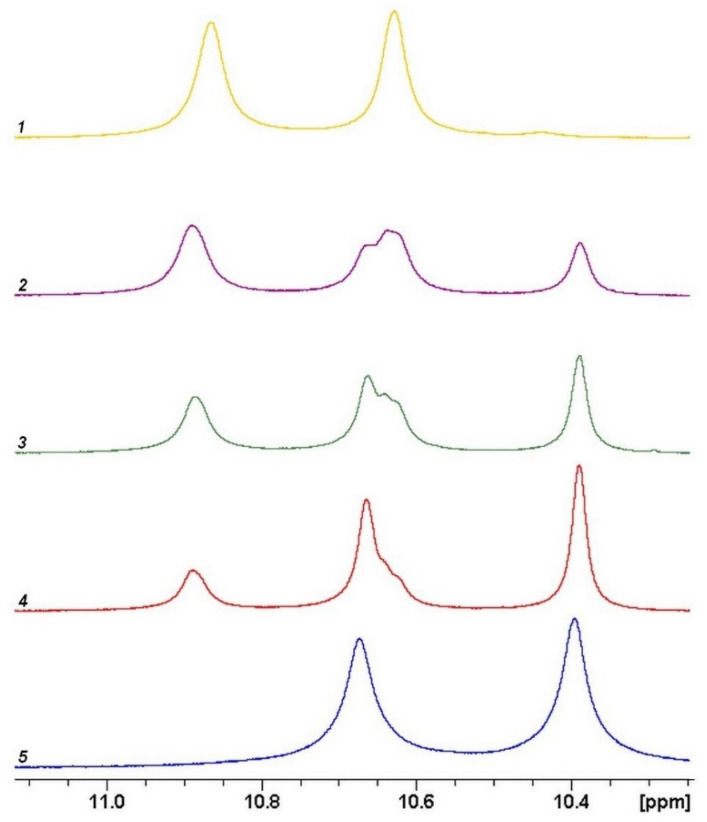
Portions of ^1^H NMR spectra of PAIs containing the signals attributed to CONH-groups (proton labels in Figure 2). (1) yellow—PAI-2, (2) violet—coPAI-3, (3) green—coPAI-2, (4) red—coPAI-1, (5) blue—PAI-1.

**^1^H NMR spectra** were recorded at 400 MHz at ambient temperature using a Bruker AC-400 spectrometer in deuterated dimethyl sulfoxide (DMSO-d_6_). Chemical shifts (δ) of spectra were reported in parts per million (ppm) based on signal of residual solvent (2.5 ppm). The results are presented in Figure 3 and Table 1.

To confirm the formation of copolymers with a given ratio of diamines, the ^1^H NMR spectra of the homo- and copolymers were analyzed (Supplementary File, Figure S1a–e). Figure 3 shows parts of spectra containing signals of NH protons of amide groups; the signals at 10.67 ppm and 10.40 ppm are assigned to PAI-1, the peaks at 10.86 ppm and 10.63 ppm are related to PAI-2. It can be seen that after increase in the DABA content in the reaction mixture, the intensity of signals at 10.86 ppm and at 10.63 ppm in the spectra of copolymers regularly increases.

The molar fraction of DABA calculated from the spectra is in good agreement with the actual loading of the components in the synthesis of copolymers (Table 1).

### 2.2. Membrane Formation

**Dense nonporous PAI (coPAI) films** were formed by the deposition in each case of a thin polymer layer from formation solutions on a glass surface using a doctor blade. Films were prepared according to the following technique. To the viscous reaction PAI (coPAI) solution an oxypropylene was added and stand to another 1 h at room temperature. This resulting 10 wt.% PAI solution was used to pour the film on to a glass support followed by removal of the solvent with heating in oven for 18 h at 50 °C, and afterwards—up to 150 °C.

The asymmetric microporous phase inversion membranes were prepared according to the dry–wet method that involved immersing the gel-film of PAI (coPAI) in N-MP on glass substrate into aqueous precipitation bath. The reaction solutions were similar to those used for preparation of dense membranes.

Similar casting conditions (temperatures, heating times, pre-casting times, precipitation times, post-treatment times, types of precipitant, concentrations and viscosities of casting solutions) were maintained for all membranes. The only variable factor was the nature of diamine component in poly(amide-imide).

### 2.3. Membrane Characterization

#### 2.3.1. FTIR Spectroscopy Study

Films of PAIs and coPAIs with ratios between components (3:7, 5:5, and 7:3), as well as asymmetric membranes based on these polymers, were studied by FTIR spectroscopy. The spectra were recorded on a Bruker Vertex 70 IR Fourier spectrometer at a resolution of 4 cm^−1^, the number of scans was 30 using a “Pike” micro-attachment with a single frustrated total internal reflection (FTIR) with a working element made of ZnSe. During registration of FTIR spectra, the correction was made that takes into account the dependence of wavelength on radiation penetration depth.

#### 2.3.2. Water and Glycerol Contact Angles of Nonporous PAI-1,2 Films

To assess adhesion characteristics of the surface (wettability, surface energy, work of adhesion), contact angles of the PAI-1 and PAI-2 samples were measured with the aid of a DSA14 device (KRṺSS) over time. The setup included: a light source, a video camera, a dosing system, and a stage for the test sample. A glass syringe (1 mL) with a metal needle 0.3 mm in diameter was used as a dispenser. The test liquids were deionized water (with a predominance of the polar component of surface tension) and glycerol (with equal polar and dispersive components).

#### 2.3.3. X-ray Diffraction Analysis

X-ray diffraction (XRD) analysis was performed at room temperature on a SEIFERT XRD 3003 TT (GE, Germany) diffractometer equipped with a primary monochromator (U = 40 kV, I = 40 mA). Cu Kα-radiation with a wavelength λ = 1.5406 Å was used. X-ray diffraction patterns were obtained with a step of 0.05° and a scanning time of 10 sec at each point of the scattering angle (2θ) region that ranged from 2° to 40°. The values of characteristic interplanar distance were calculated using Bragg’s equation [23].

#### 2.3.4. Electron Microscopy Study

Membrane morphology was studied by scanning electron microscopy (SEM) using a JSM-35C instrument (Jeol, Japan). Before the study, gold layers 20 nm thick were deposited by thermal vacuum deposition onto the surface of low-temperature cleavages.

#### 2.3.5. Atomic Force Microscopy (AFM) Study

AFM studies of the studied samples were performed with the aid of a Nanotop NT-206 atomic-force microscope (ODO “Microtestmachines”, Gomel, Belarus) in the contact and tapping modes under atmospheric conditions using FMG01silicon cantilevers with a force constant 1–5 N/m and a tip curvature radius of 10 nm. The experimental data were processed using the Surface Explorer program.

Calculation formulas of *Ra* (arithmetic mean surface roughness) and *Rq* (rms surface roughness) are shown below:

(2) $$R_{a}=\frac{1}{N}\sum_{n=1}^{N}\left|r_{n}\right|$$

(3) $$R_{q}=\sqrt{\frac{1}{N}}\sum_{n=1}^{N}r_{n}^{2}$$

where *r_n_* is the height of the *n*th point above the midline, *N* is the total number of points.

#### 2.3.6. Thermal Analysis

Thermal analysis of samples was performed with a 204 F1 differential scanning calorimeter (NETZSCH, Selb, Germany) in argon atmosphere, in the temperature range from ambient temperature to 350 °C; the heating rate was 10 °C·min^−1^. The argon flow rate was 25 mL·min^−1^, the gas flow rate intended for cooling the measuring chamber was 70 mL·min^−1^. Thermal gravimetric analysis was performed using a Netzsch TG 209 F1 instrument in the temperature range from ambient temperature to 600 °C at a heating rate of 10 °C·min^−1^ in the inert gas flow. The weight of a tested sample was approximately 2–3 mg.

#### 2.3.7. Transport Measurements

Permeate water flow through a porous membrane *Q* [kg·m^−2^·h^−1^·bar^−1^] was determined by filtration experiments in dead-end mode in an ultrafiltration cell (Amicon Millipore, Bedford, MA, USA) with a membrane area of 3.14 × 10^−4^ m^2^. The transmembrane pressure *Dp* [bar] was kept constant at a selected value from the range of 1–4 bar (radial pressure gauge, WATTS, USA) using a compressed nitrogen cylinder connected to the filter cell.

(4) $$Q=m\times S^{-2}\times t^{-1}\times D_{p}{}^{-1}$$

where *m* [kg] is the weight of permeate; *S* (m^2^) is the membrane sample area, *t* [h]—the outflow time.

**Figure 4:**
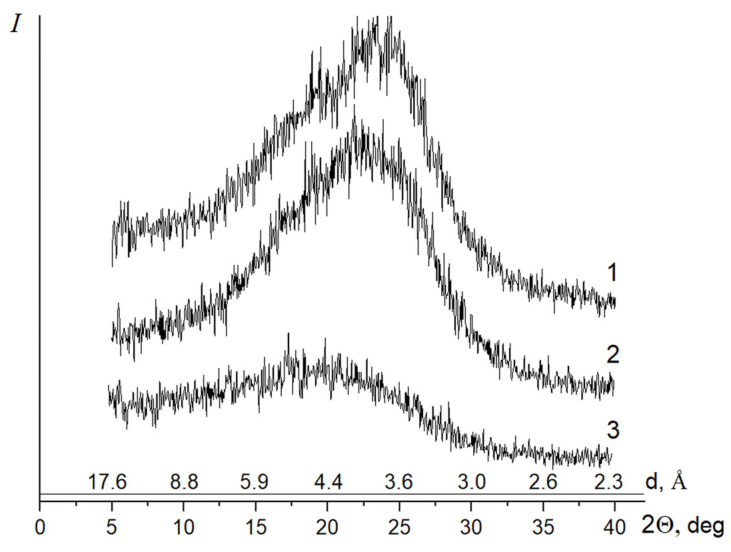
Diffraction patterns of coPAI-3 (curve 1), coPAI-2 (curve 2), and coPAI-1 (curve 3) with different ratios of PAI-1 and PAI-2 components.

Pervaporation properties of the obtained dense membranes (nonporous films) were tested for different penetrants (water, ethanol, methanol, cyclohexane) using a non-continuous flow laboratory cell, as described in ([21], Figure 4a) with an operating membrane area of 1.38 × 10^−3^ m^2^ at a constant temperature of 40 °C. Permeate vapors were condensed using liquid nitrogen. The received permeate was weighed and the flux value J [kg⋅m^−2^⋅h^−1^] was estimated using the following equation:

(5) $$J=m\times S^{-2}\times t^{-1}$$

where *m* is the mass [kg] of the penetrant permeated through membrane area *S* [m^−2^] in a period *t* [h^−1^]; *P* = *J*·*l* [μm⋅kg⋅m^−2^⋅h^−1^] (permeation rate) is the flux of a penetrant normalized to the membrane thickness of 1 μm.

## 3. Results and Discussion

In the present work, new co-poly(amide-imide)s with the general structural formula shown in Figure 2 were synthesized. The prepared polymer homologs differed in the content of repeating units containing diaminobenzoic acid fragments. Although the monomers used in the polycondensation process are known to have similar reactivities, the structure and precise composition of the formed products are still unclear. From general considerations, it is expected that they are random copolymers with different contents of competing diamine fragments. In our work, the exact compositions of the copolymers were found using the NMR spectroscopy data; the results are presented in Table 1.

Various types of samples (powders, non-porous films, asymmetric porous membranes of complex morphologies) were obtained from all synthesized polymers. Since the same starting solutions were used in preparation of samples of different types, it became possible to perform comparative analysis of polymers and to reveal the contribution of carboxyl-containing fragments to structural and transport characteristics of the produced membranes.

Thermal properties of the studied co-poly(amide-imide)s determined by thermal gravimetric analysis are illustrated in Figure 1 and presented in Table 2. The initial weight loss of the samples in the temperature range from room temperature to 150 °C is associated with the release of adsorbed water; the temperature of the maximum rate of this process (T_1_) was determined from the DTG data (see Table 2). In the temperature range from 150 °C to 320 °C, the release of free solvent (N-MP) from co-poly(amide-imide) films is mainly observed (the boiling temperature of N-MP is 202 °C). This process is accompanied by liberation of water bound to polymers by hydrogen bonds. The temperatures of the maximum rate of release of the solvent from the copolymers in this temperature range (T_2_) are also presented in Table 2. Note that the temperature of the maximum rate of this process increases with increasing the PAI-1 content in the copolymers. This unexpected result requires detailed study using additional physico-chemical methods.

Heating the samples at temperatures from 350 °C to 450 °C leads to further weight loss (up to 8 wt.%). At this stage, in addition to water and the solvent, carbon dioxide, N-MP/water complexes and an excess of diaminobenzoic acid are released. The temperatures of the maximum rate of this process (the release of the bound solvent (T_3_) are given in Table 1 and indicated on the DTG curves in Figure S2 (see Supplementary File). The regular increase in the T_3_ temperature with an increase in the percentage of DABA in copolymers is observed, since carboxyl groups of DABA can form complexes with N-MP. Above 400 °C, chain degradation (decomposition of amide groups) begins. The temperature of the maximum rate of thermal destruction of the coPAI main chain (T_4_) is also given in Table 1 and correlates with the content of the hard PAI-2 phase in copolymers.

Glass transition temperatures of coPAI were determined by DSC. Thermograms of the first and second scans are shown in Figure S3a–c (see Supplementary File). It is seen that during the first scan up to 400 °C, two endotherms are recorded for all coPAI samples. Low-temperature endotherms reflect the release of free water from coPAI, high-temperature endotherms are related to the release of residual solvent (N-MP). The temperatures of the maximum of the low-temperature endotherm correlate with the number of hydrophilic groups, which increases with an increase in the DABA content in coPAI. The temperatures of the maximum endotherm of the N-MP release practically coincide.

During the second scanning up to 400 °C, the glass transition step is recorded for all three samples of coPAI. The glass transition temperatures of the samples, as well as their thermal stability, increase with an increase in the content of more rigid PAI-2 phase in the copolymer.

Curve 1 in Figure 4 shows the diffraction pattern of the film containing the PAI-1 and PAI-2 components in the 3:7 ratio. Curve 2 is the diffraction pattern of the film that contains the PAI-1 and PAI-2 components in the 5:5 ratio. Curve 3 (Figure 4) shows the diffraction pattern of the film of the PAI-1 and PAI-2 in the 7:3 ratio.

Diffraction pattern 1 (the sample with the maximum PAI-2 content in the copolymer) exhibits the amorphous halo at 2θ = 19.30° and reflections at 2θ = 16.30 and 24.0°, which correspond to interplanar spacings of 5.44 and 3.70 Å.

The pattern is similar in the case of the (5:5) copolymer. The position of the maximum of the amorphous halo remains the same. The reflexes at 2θ = 16.30 and 24.0° are also registered.

The diffraction pattern of the sample with the minimum PAI-2 content in the copolymer shows only the amorphous halo with a maximum at 2θ = 18.0°.

**Figure 5:**
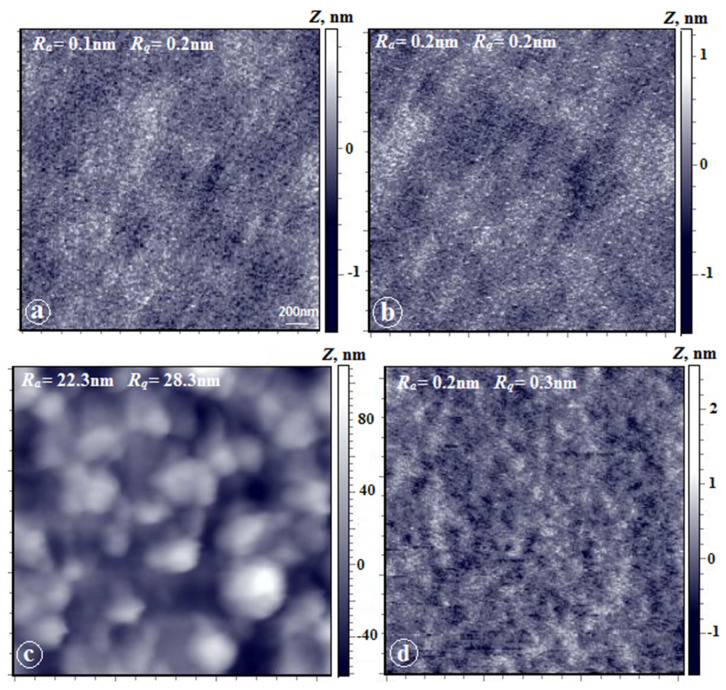

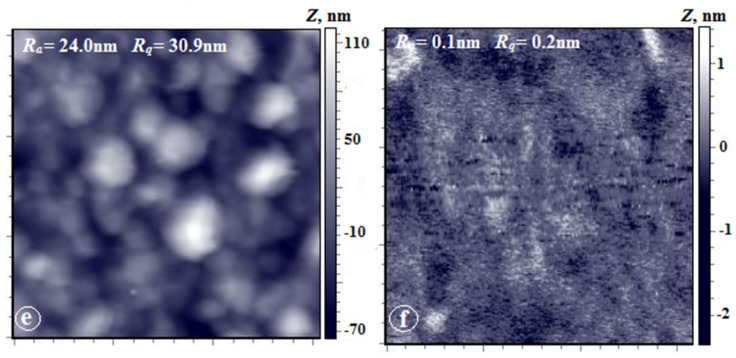
AFM height images of film surfaces: coPAI-1 (**a**,**b**), coPAI-2 (**c**,**d**) and coPAI-3 (**e**,**f**). Scan matrix size—3 × 3 µm; (**a**,**c**,**e**) —the upper surface, (**b**,**d**,**f**)—the substrate-side surface.

Figure 5 shows AFM images of the upper surface and the substrate-side surface of coPAI (7:3), coPAI (5:5), and coPAI (3:7) film samples. As can be seen, morphologies of both surfaces (Figure 5a,b) are almost identical for the (7:3) ratio; they demonstrate extremely low values of the arithmetic mean (Ra) and rms (Rq) surface roughnesses (0.2 nm and 0.3 nm), which has been previously observed for the upper surfaces of PAI-1 films [8]. This is a fine-grained nanoporous surface. On both surfaces, the grains are oriented along the film plane, which is also typical of upper surfaces of the PAI-1 homopolymer films [8].

The morphology of the surfaces of the coPAI-2 films (Figure 5c,d) differs significantly from that of the coPAI-1 samples. On the upper surface, formations close to spherical (domains) characteristic of the morphology of the upper surface of nonporous PAI-2 films [8,23] are observed. The domain size varies from 100 to 300 nm; individual domains with sizes up to 500 nm also visible. The roughness parameter increases by two orders of magnitude (Ra = 22 nm and Rq = 28 nm). The substrate-side surface (Figure 5d) shows low values of the roughness parameter and is morphologically identical to that of the (7:3) PAI films. The peculiar feature of this film is the difference between the morphologies of the substrate-side surface and the upper surface that have radically different roughness parameters.

The upper surface of the coPAI-1 film (Figure 5e) also has domain morphology with slightly higher roughness parameters (Ra = 24 nm, Rq = 30 nm). The most interesting results were obtained by AFM studies of the substrate-side surfaces of coPAI-3 films (Figure 5f). The domains identical to those observed on the upper surface are visualized under the difficult-to-determine upper layer, which necessitates analysis of phase contrast of this image.

**Figure 6:**
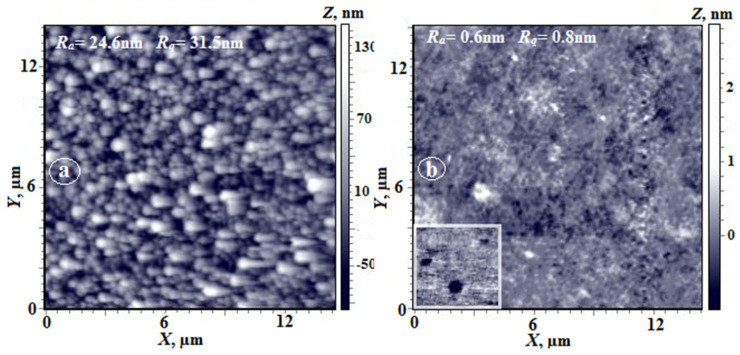
AFM height image of surface of film coPAI-3; (**a**,**b**). (**a**)—the upper surface, (**b**)—the substrate-side surface. The inset in Figure 6b presents a contrast of lateral forces.

The corresponding images of the surfaces of the coPAI-1 film (scanning area: 142 microns) are shown in Figure 6. The domain structure typical of this film is observed on the upper surface (Figure 6a). The spherical formations similar to the domains on the upper surface are also clearly visible on the substrate-side surface (Figure 6b). Analysis of the AFM image obtained in the mode of contrast of lateral forces simultaneously with the image of topography (see the inset in Figure 6b) allowed us to conclude that in this case, a two-phase system is observed, with phases identical for homopolymers PAI-1 and PAI-2.

Table 3 shows characteristics of the upper and lower surfaces of PAI-1 and PAI-2 non-porous films obtained under the same conditions. It is shown that in the case of PAI-1, the upper and lower surfaces have approximately the same energies, and in the case of PAI-2, the values differ significantly. Moreover, the total value of the surface energy on the side of the film where intensive evaporation of the solvent took place, is significantly lower. In both cases, the dispersion component of the free surface energy is significantly higher on the upper side and, therefore, the polar component is higher on the side contacting with glass. In the case of PAI-2, this effect manifests itself most clearly, apparently, due to the presence of carboxyl groups oriented toward the lower surface, which was formed during contact with hydroxyl-containing glass.

The dense films (nonporous membranes), whose properties were discussed above, were obtained by free evaporation of the solvent from surfaces; the evaporation conditions were the same for all samples. The starting solutions were also the same (their preparation is described in detail in the Experimental section). The same starting solutions were used to prepare phase inversion membranes, which were obtained under similar wet spinning conditions (water precipitation bath, time and temperature, settling time and drying conditions, post-processing technology).

**Figure 7:**
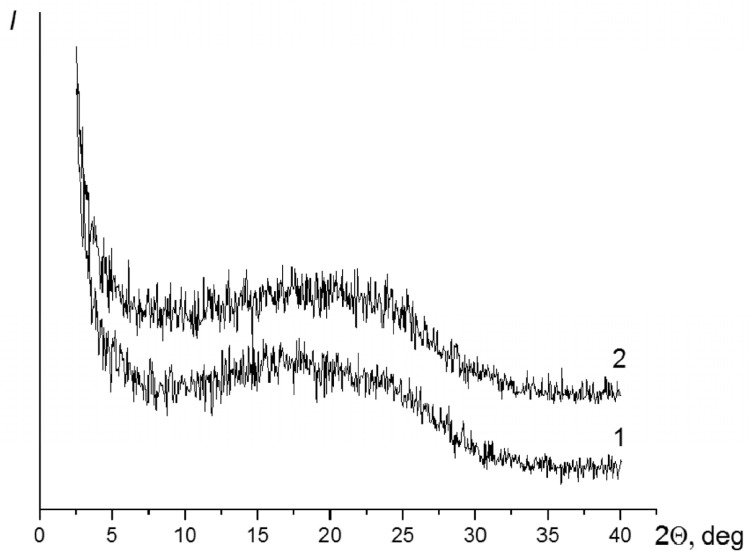
Diffraction patterns of the coPAI-2 membrane, taken from the side of the glance layer (1) and from the matte side (2).

All the resulting asymmetric membranes were amorphous porous films; the example of coPAI-2 illustrates their structure (Figure 7).

**Figure 8:**
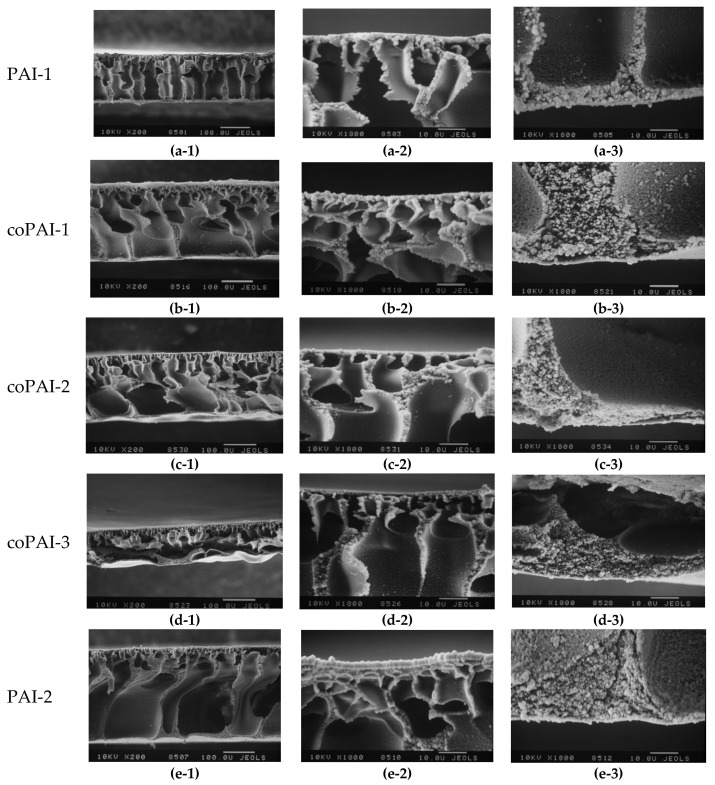
SEM images of low-temperature fractures of asymmetric membranes PAI-1(0% DABA; **a-1**,**a-2**,**a-3**), coPAI-1(30% DABA; **b-1**,**b-2**,**b-3**), coPAI-2 (50% DABA; **c-1**,**c-2**,**c-3**), coPAI-3 (70% DABA; **d-1**,**d-2**,**d-3**), and PAI-2 (100% DABA; **e-1**,**e-2**,**e-3**). (**a-1**,**b-1**,**c-1**,**d-1**,**e-1**)—general cross-sectional view; (**a-2**,**b-2**,**c-2**,**d-2**,**e-2**)—top cross-sectional view; (**a-3**,**b-3**,**c-3**,**d-3**,**e-3**)—bottom cross-sectional view.

Figure 8 shows the results of scanning electron microscopy studies of the morphology of asymmetric membranes obtained under the same conditions. Comparative analysis of cross-sectional images of asymmetric membranes shows that introduction of diaminobenzoic acid fragments strongly affects morphology of the membranes formed in aqueous precipitation bath.

Since all the membranes were obtained under equal conditions, the only factor that could affect the formation process was chemical structure of the polymer. For a series of polymers PAI-1, coPAI-1, coPAI-2, coPAI-3, and PAI-2, the same precipitation bath gradually became “softer”. Therefore, the upper skin layer and macropore walls should become thicker, and a large number of small pores should appear in the sublayer of the asymmetric membrane. This trend is clearly visible when homopolymers are compared. However, in the case of copolymers, the SEM images show individual morphological features in the skin region of each membrane. Moreover, in going from coPAI-1 to coPAI-3, the skin layer apparently becomes thinner and denser.

This effect can possibly be related to inhomogeneous distribution of various fragments of polymer chains in gel films of copolymers at the stage of preforming before deposition into precipitation bath. Thus, the detailed study of morphology of the surfaces of asymmetric membranes by atomic force microscopy is necessary.

**Figure 9:**
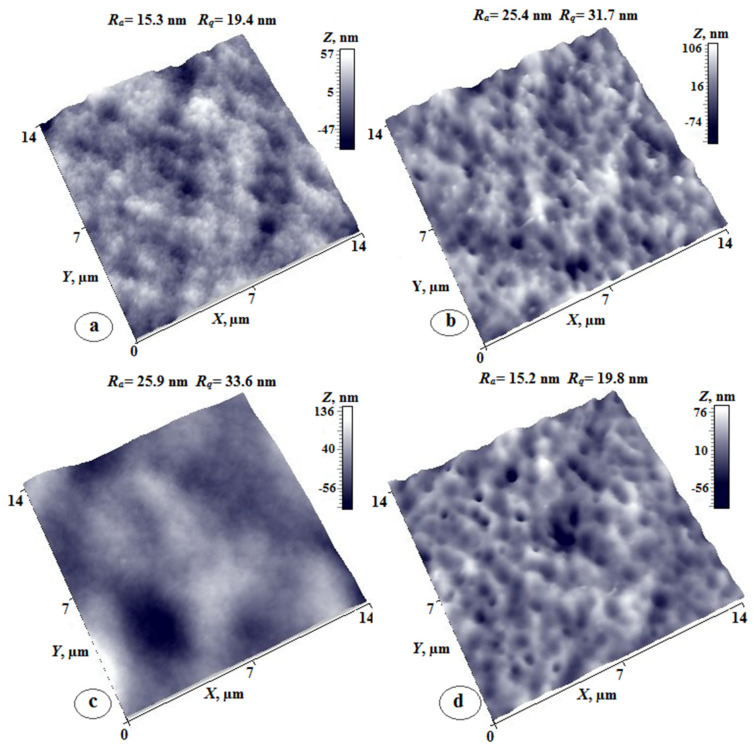
3D AFM images of surfaces of the coPAI-1 (**a**,**b**), coPAI-2 (**c**,**d**) and coPAI-3 (**e**,**f**) membranes; (**a**,**c**,**e**)—the upper surface, (**b**,**d**,**f**)—the substrate-side surface.

The surfaces of co-poly(amide-imide) membranes prepared by the phase-inversion process were also investigated by AFM. The main feature of the obtained membranes is the uniform morphology of the substrate-side surface (“the matte surface”, Figure 9b,d,f) with characteristic crater-like formations and non-monotonic dependence of roughness parameters on copolymer composition.

Thus, the values of the arithmetic mean (Ra) and rms (Rq) surface roughness for co-poly(amide-imide)s PAI-2 (30, 50, and 70%) are equal to 25, 15, 21 nm and 31, 19, 28 nm, respectively.

Figure 9a,c,e shows three-dimensional images of membranes from the skin layer (Gloss), which are characterized by the developed complex nano-domain morphology with pores whose sizes differ significantly depending on the content of PAI-2 in the copolymer. Thus, the average pore sizes for the (7:3) coPAI vary from 300 nm to 1.5 microns, while for the (3:7) coPAI, pore size increases slightly and reaches a maximum value of 2 microns. This membrane has more homogeneous morphology.

The sizes of the pores in the (5:5) coPAI on the skin layer side are the largest (Figure 9c) and reach 4 microns. The height of the profile for this area of the surface is ~140 nm. At the same time, the surface roughness parameters of the skin layer side, as well as those of the substrate-side surface, change non-monotonically: the maximum values of Ra and Rq (26 and 34 nm) were observed for the (5:5) coPAI.

The values of the roughness of the skin layer surface for other two coPAIs differ slightly; the minimum values obtained for the (3:7) coPAI (the Ra and Rq values were equal to 15 and 19 nm, respectively). The thin membrane structure on the skin layer side has a nano-domain, nano-porous morphology, which is clearly observed in the case of the membrane based on the (3:7) coPAI.

**Figure 10:**
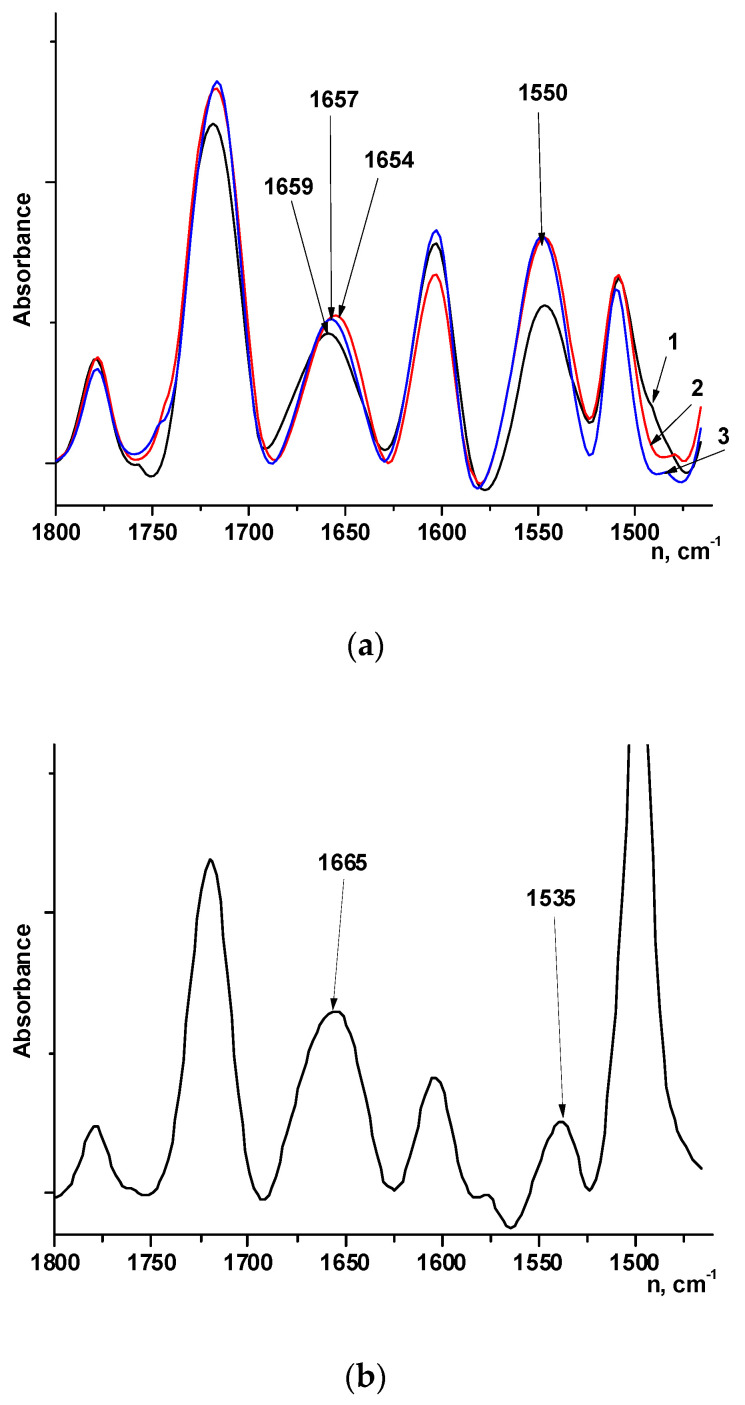

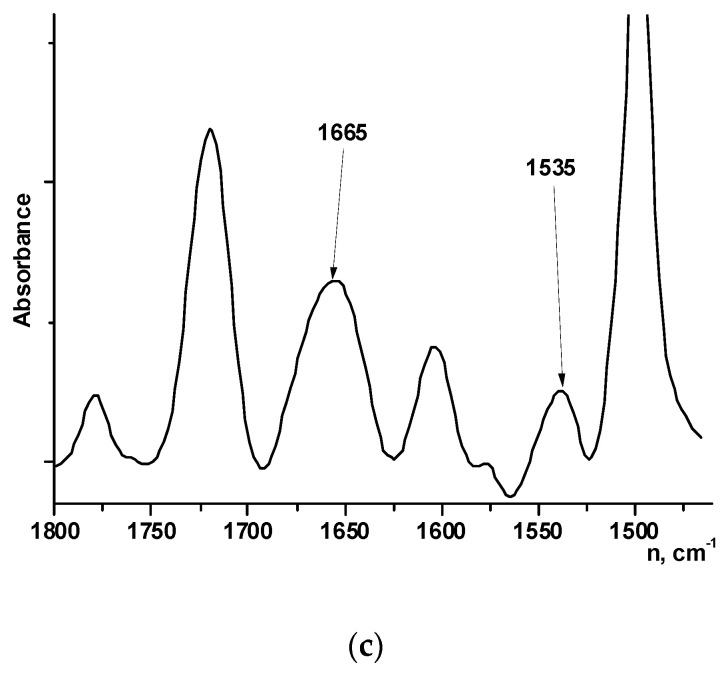
FTIR spectra of (**a**) PAI-2: (1) powder, (2) film, (3) asymmetric membrane; (**b**) PAI-1; (**c**) coPAI-2 (50% DABA): (1) asymmetric membrane, (2) film.

The films of PAIs and copolymers with different component ratios (3:7, 5:5, and 7:3) were studied by IR spectroscopy (Figure 10a–c). Phase-inversion films (asymmetric membranes) of coPAIs with different ratios between components (3:7, 5:5, and 7:3) were also investigated by FTIR spectroscopy.

Analysis of the PAI-2 spectra in the 1800–1450 cm^−1^ region (Figure 10a) shows the presence of the absorption bands characteristic of PAI (1780 cm^−1^, 1720 cm^−1^, 1655 cm^−1^, and 1550 cm^−1^). However, the intensity of the 1550 cm^−1^ band is relatively high in comparison with that of the 1655 cm^−1^ band (compare the ratio between intensities of these peaks in the PAI-1 spectrum, Figure 10b).

Such an increase in the intensity of the 1550 cm^−1^ band may be related to the fact that this band is complex and consists of the sum of the absorption bands of bending vibrations of NH group and anti-symmetric vibrations of the ionized carboxyl group COO^−^. Ionization of carboxyl group can occur due to interaction between carboxyl group COOH and the NH group of the amide. The spectrum of PAI-2 in the powder form (1) contains a shoulder near 1685 cm^–1^ against the background of the band at 1659 cm^−1^, which indicates the presence of non-ionized COOH groups. In going to films prepared from solutions in amide solvents, the shoulder near 1685 cm^−1^ disappears, and intensity of the COO^−^ band increases (Figure 10a, (2)—PAI-2 film, (3)—asymmetric PAI-2 membrane), which is possible when COOH groups interact with the solvent.

Comparison of IR spectra of the coPAI-2 film and asymmetric membrane (Figure 10c) shows that ratios between intensities of absorption bands assigned to different fragments of the copolymer (imide units (1780 cm^−1^, 1720 cm^−1^), amide fragments (1650 cm^−1^ and 1550 cm^−1^), in-plane vibrations of benzene rings in the areas of 1600 cm^−1^ and 1500 cm^−1^) are different for these two types of samples.

This difference is probably caused by different morphologies of asymmetric membrane and film, which, in turn, depend on preparation method, and, possibly, by different solubilities of PAI-2 and PAI-1 fragments in the used solvents (water and N-MP).

**Figure 11:**
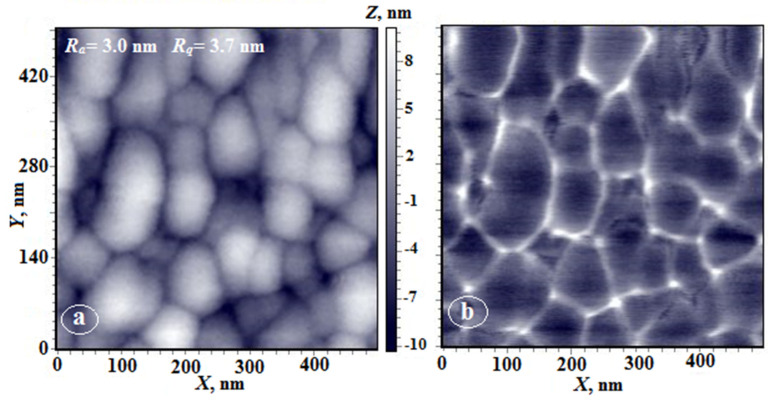
AFM images of the free (upper) surface of PAI-2 film (**a**,**b**); (**a**)—height image, (**b**)—phase contrast image.

It can be assumed that the presence of residual solvent that is strongly bound to polymer fragments containing DABA carboxyl groups has a significant effect on conformational mobility of polymer chains, which manifests itself in the morphology of both nonporous and porous films. This assumption is confirmed by the AFM images presented in Figure 11, which clearly illustrate the effect.

Due to high hydrophilicity of the PAI-2 homopolymer, surface morphology of its nonporous films depends on the rate of solvent release during film preparation. Figure 11 shows images of the upper surface of the film (height and phase contrast), from which the solvent was released slowly. As is seen, the morphology differs significantly from that shown in Figure 1c. In Figure 11, domains with an average longitudinal size of 100 nm are visualized. Note their mutual orientation in the plane in the direction that is apparently related to the direction of movement of the doctor blade during film preparation. In the phase contrast mode (Figure 11b), two phases are clearly visible: the polymer phase and the solvent, which covers each domain. According to thermogravimetric analysis, the amount of residual solvent reaches 10 wt.% [24].

**Figure 12:**
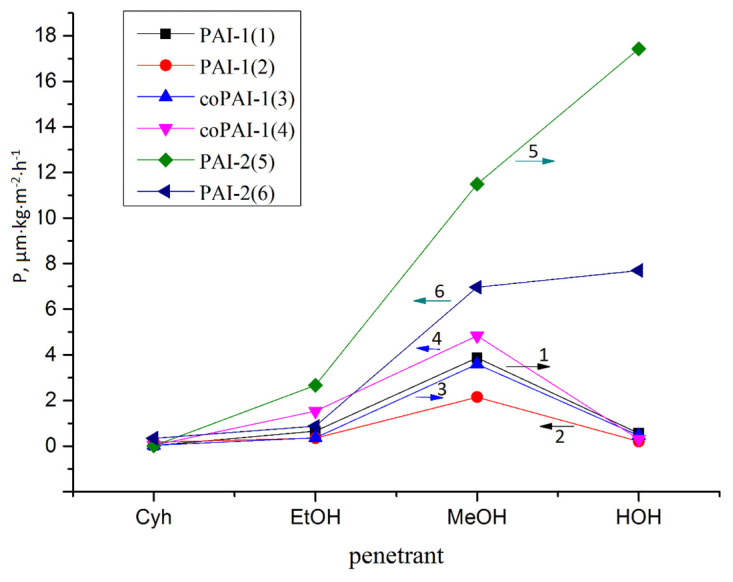
Diagram (general view) of the permeability rate values (P [µm⋅kg⋅m^−2^⋅h^−1^]) of dense membranes (nonporous films) of the synthesized polymers PAI-1 (**1**,**2**), coPAI-1 (**3**,**4**), PAI-2 (**5**,**6**) in relation to penetrants of different polarities (Cyh—cyclohexane, EtOH—ethanol, MeOH—methanol, HOH—water). The mean values are shown, n ≥ 3. The order of penetrants during pervaporation: direction 1 (→ arrow on the diagram) Cyh → EtOH → MeOH → HOH; direction 2 (← arrow on the diagram) HOH → MeOH → EtOH → Cyh.

Transport properties of the obtained polymers correlate well with the above results concerning surface characteristics of membranes, their domain structure, and the presence of strongly bound residual solvent. The results presented in Figure 12 and Table 4 clearly demonstrate that all the studied membranes are stable under pervaporation conditions. The flow of cyclohexane through these films practically does not change after a cycle of pervaporation experiments, in which polar liquids are involved. After each measurement, the membranes were dried to such an extent that when the dry membrane was placed in the pervaporation cell and evacuated, the residual pressure under the membrane was minimal and stable (test for residual unbound solvent and membrane integrity).

Analysis of the presented data shows that the permeability for cyclohexane is practically similar for all the studied membranes. The same is true about the water permeate rates of PAI-1 and coPAI-1. The fluxes of PAI-1 and coPAI-1 for all other penetrants within the measurement error are practically the same in the first part of the test cycle. Naturally, the level of permeability of PAI-2, which contains carboxyl groups in each repeating unit, with respect to polar penetrants significantly exceeds those of other polymers. The water transport through this polymer is especially striking. Nevertheless, after the cycle of pervaporation of polar liquids, the flux of cyclohexane through the PAI-2 film is similar to that measured at the beginning of pervaporation. Thus, the flow of water and alcohols through the membrane apparently does not cause any significant changes in its composition and structure.

It is interesting to note that coPAI-1 in the first part of the cycle of pervaporation experiments behaves like PAI-1. At first glance, this seems strange, since the introduction of more rigid fragments into the macromolecule is accompanied by an increase in Tg, as is discussed above in the description of the thermal properties of the studied co-poly(amide-imide)s. This effect usually leads to a decrease in the diffusion permeability of non-porous membranes due to a decrease in the mobility of molecular chains ([25], n 4.3.5). However, in the case of the copolymers studied in this work, there are additional factors that affect the transport properties of membranes. In the second part of the cycle shown in Figure 12 coPAI-1 is “under the influence” of PAI-2. At the same time, in the second part of the cycle, the flux of methanol through PAI-1 decreases, while the methanol flux through coPAI-1 increases. This effect may be related to the domain structure of polymers, which is more pronounced in PAI-2, but is also present in copolymers.

This structural feature was revealed to be evident in the properties of asymmetric membranes, which in dense skin layers have characteristic surface irregularities, caverns and large domain-like formations. The resulting asymmetric membranes are distinguished by their permeability to water. The flow of water through the membranes under the conditions of the ultrafiltration cell for coPAI-1 and coPAI-3 Q = (2 ÷ 10) kg⋅m^−2^⋅h^−1^⋅bar^−1^, while for the case of coPAI-2 the Q value is greater than 50 kg⋅m^−2^⋅h^−1^⋅bar^−1^. The presented results will stimulate further research in this area, since there is high demand for membranes capable of screening out nano-scale negatively charged biologically active molecules. It is also interesting to find methods that will permit finer control of the structure of asymmetric and non-porous poly(amide-imide) membranes.

## 4. Conclusions

New copolymers of aromatic poly(amide-imide)s containing different amounts of carboxyl-containing fragments were synthesized by low-temperature polycondensation in solution. The dense nonporous and asymmetric porous membranes obtained from the synthesized polymers have complex morphologies, whose appearance is caused by the presence of residual solvent strongly bound to the polymer; this factor affects the film formation process. The presence of the solvent in the polymer matrix exerts direct influence on the pervaporation transport of polar liquids across membranes (since it mixes well with these solvents). The residual solvent also indirectly determines morphology of the domain-like skin layer of asymmetric membranes. Although it is obvious that use of coPAI copolymers instead of PAI-1 and PAI-2 makes it possible to obtain materials with unique properties, it is necessary to carry out further research work, including the study of behavior of copolymers in solutions (with the purpose of revealing the most significant factors that influence and control membrane formation processes).

## Figures and Tables

**Table 1:** Composition of copolymers.

PAI	Ratio of Components (% of DABA)	
	Loaded Into the Reaction Mixture (Diamine Percentage)	Calculated from ^1^H NMR Spectra
coPAI-1	30	32
coPAI-2	50	49
coPAI-3	70	68

**Table 2:** Thermal properties of coPAIs samples.

Sample	% of DABA	T_1_, °C	T_2_, °C	T_3_, °C	T_g_, °C	T_4_, °C	Carbon Yield at 600 °C, %
coPAI-1	30	78.6	237.7	420.1	345.5	497.7	39.5
coPAI-2	50	66.9	224.7	425.0	352.3	542.2	43.0
coPAI-3	70	78.1	223.9	433.1	361.3	548.3	45.7

T_1_—the temperature of the maximum rate of release of adsorbed water; T_2_—the temperature of the maximum rate of release of the free residual N-MP solvent in the 180–300 °C temperature range; T_3_—the temperature of the maximum rate of release of the residual N-MP solvent bound to the polymer in the 300–440 °C temperature range; T_4_—temperature of the maximum rate of thermal destruction of coPAI samples; T_g_—glass transition temperatures of coPAIs.

**Table 3:** Surface characteristics of dense films (1—upper surface, 2—lower surface).

			Contact Angle, (°)				Surface energy, mJ⋅m^−2^		
№	Sample		Water		Glycerol		Total	Dispersion Component	Polar Component
				∆		∆			
1	PAI-1	1	78.01	3.01	68.01	1.00	30.30	19.91	10.39
		2	75.00		67.01		30.40	16.60	13.80
2	PAI-2	1	83.43	12.76	70.77	6.42	29.23	22.63	6.61
		2	70.67		64.35		33.09	15.05	18.05

**Table 4:** Pervaporation properties (P [µm⋅kg⋅m^−2^⋅h^−1^]) of dense membranes at 40 °C. The order of penetrants during pervaporation: (1) Cyh → EtOH → MeOH → HOH; (2) HOH → MeOH → EtOH → Cyh.

	PAI-1 (1)	PAI-1 (2)	coPAI-1 (1)	coPAI-1 (2)	PAI-2 (1)	PAI-2(2)
*l*, μm	19	19	22	22	21	21
Cyh	0.022 ± 0.001	0.164 ± 0.008	0.030 ± 0.002	0.030 ± 0.002	0.014 ± 0.001	0.342 ± 0.017
EtOH	0.658 ± 0.033	0.348 ± 0.017	0.367 ± 0.018	1.535 ± 0.077	2.663 ± 0.133	0.869 ± 0.043
MeOH	3.869 ± 0.193	2.141 ± 0.107	3.589 ± 0.179	4.837 ± 0.242	11.493 ± 0.575	6.966 ± 0.348
HOH	0.551 ± 0.028	0.199 ± 0.010	0.459 ± 0.023	0.299 ± 0.015	17.425 ± 0.871	7.700 ± 0.385

## Data Availability

Data is contained within the article or Supplementary Materials.

## Supplementary Materials

The following are available online at https://www.mdpi.com/article/10.3390/membranes12010091/s1, Figure S1: a. 1H NMR spectrum of PAI-1; b. 1H NMR spectrum of PAI-2; c. 1H NMR spectrum of coPAI-1; d. 1H NMR spectrum of coPAI-2; e. 1H NMR spectrum of coPAI-3; Figure S2: TG (solid lines) and DTG (dashed lines) curves of coPAI-1 (green), coPAI-2 (brown), coPAI-3 (blue) ; Figure S3: a. 1-st and 2-nd DSC scan curves of coPAI-1; b. 1-st and 2-nd DSC scan curves of coPAI-2; c. 1-st and 2-nd DSC scan curves of coPAI-3.
