# Supplementary material for: New Membrane-Forming Aromatic Co-Poly(amide-imide)s: Influence of the Chemical Structure on the Morphological, Thermal and Transport Properties
Source: Membranes (Basel). 2022 Jan 14;12(1):91. doi: 10.3390/membranes12010091 (PMC8781751; doi:10.3390/membranes12010091)
Supplement: Supplementary file 1 [file membranes-12-00091-s001.zip › membranes-1519472-supplementary.pdf]

Supplementary Materials

New Membrane-Forming Aromatic Co-poly(Amide-Imide)s. Influence of the Chemical Structure on the Morphological, Thermal and Transport Properties

Svetlana V. Kononova*, Danila A. Kuznetsov, Galina N. Gubanova, Elena V. Kruchinina, Anatoly Ya. Volkov, Milana E. Vylegzhanina, Elena N. Vlasova and Boris Z. Volchek

Institute of Macromolecular Compounds Russian Academy of Science, 199004, Bolshoy pr. 31, Saint-Petersburg, Russia

* Correspondence: svetlanavkononova@gmail.com (S.V.K.)

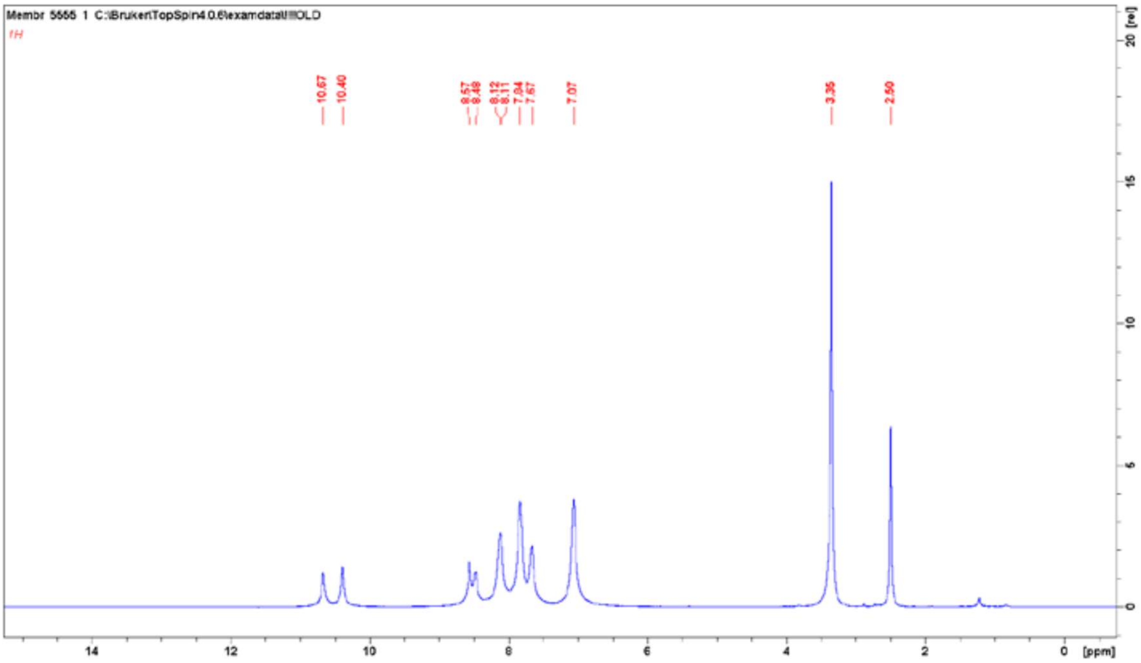

Figure S1a. 1H NMR spectrum of PAI-1.

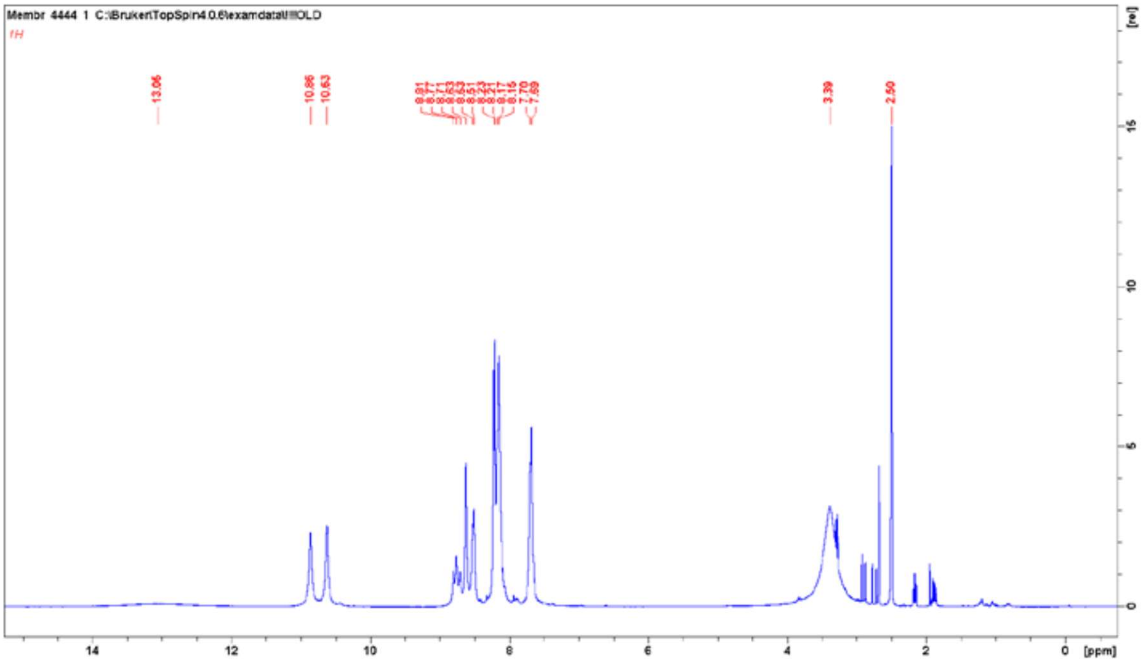

Figure S1b. 1H NMR spectrum of PAI-2.

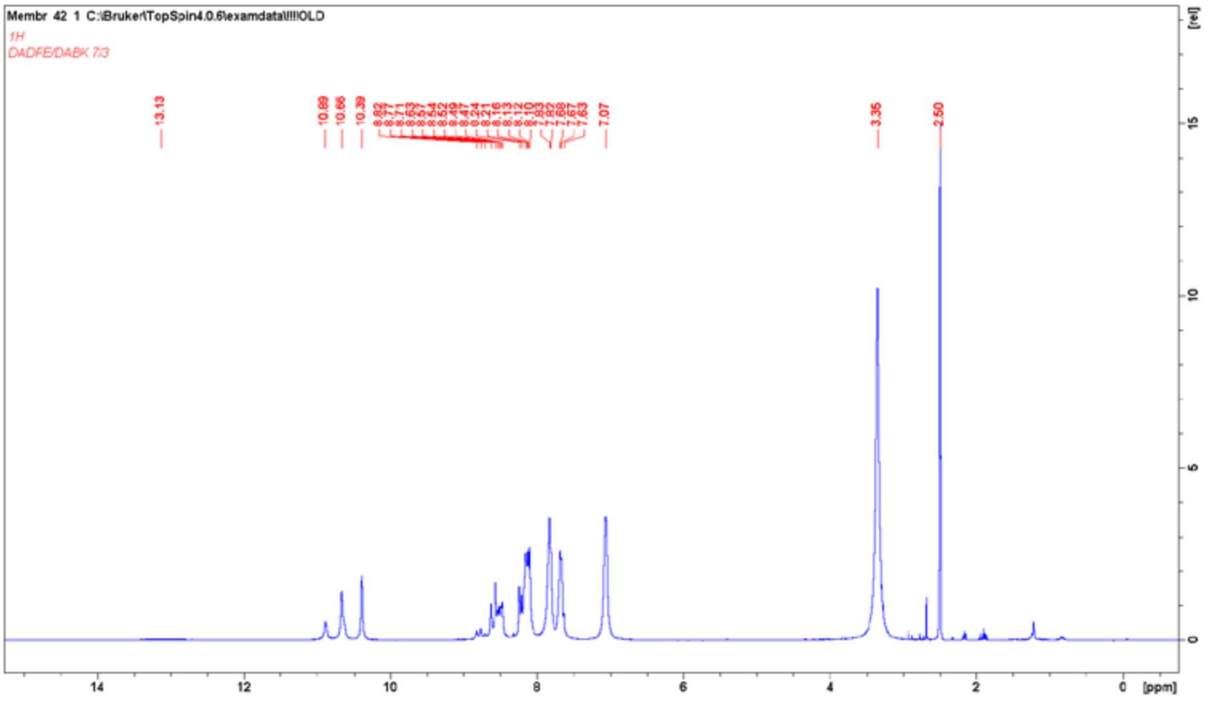

Figure S1c. 1H NMR spectrum of coPAI-1.

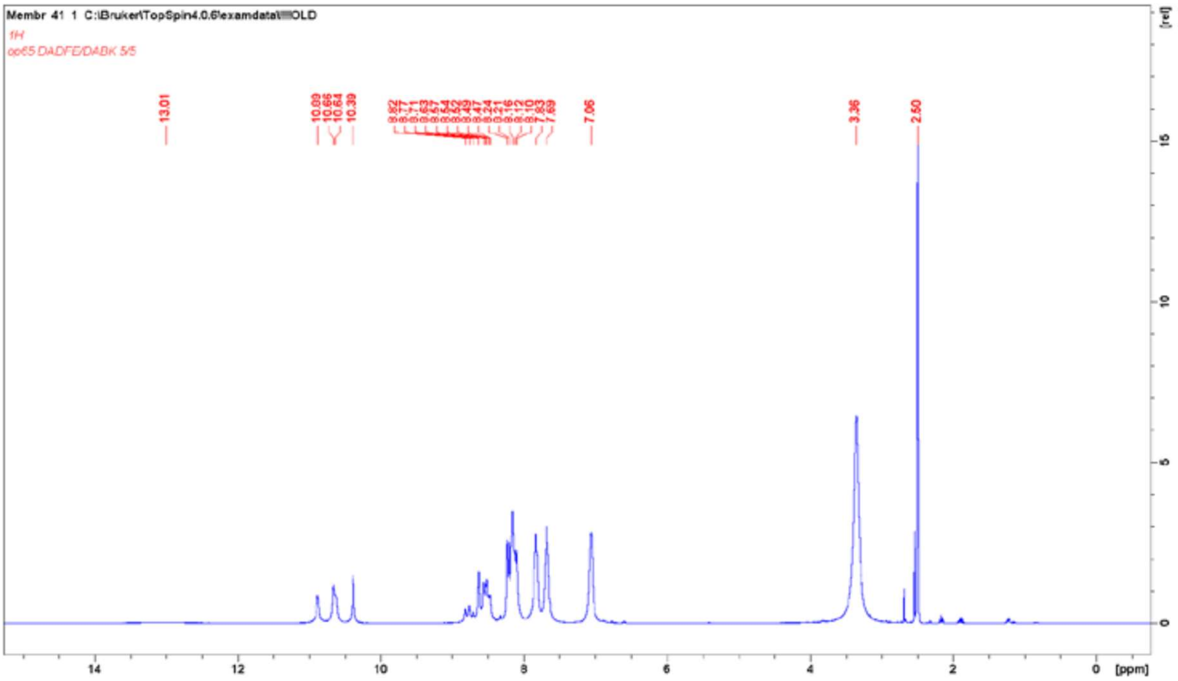

Figure S1d. 1H NMR spectrum of coPAI-2.

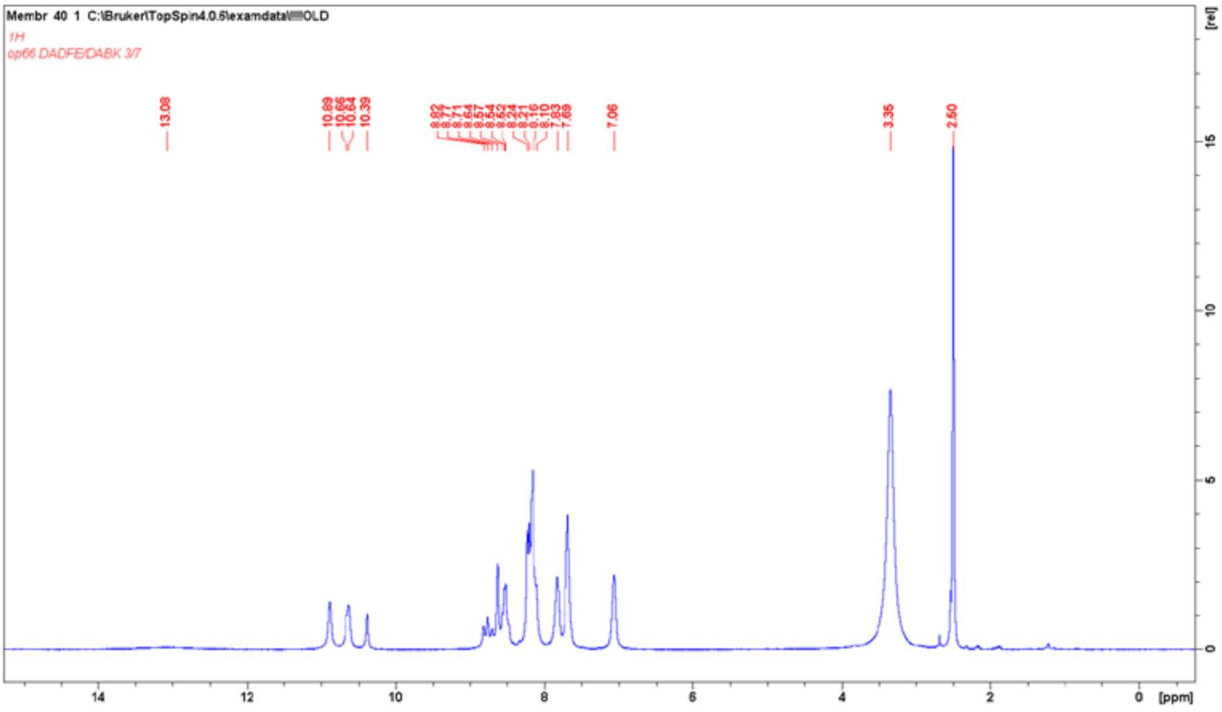

Figure S1e. 1H NMR spectrum of coPAI-3.

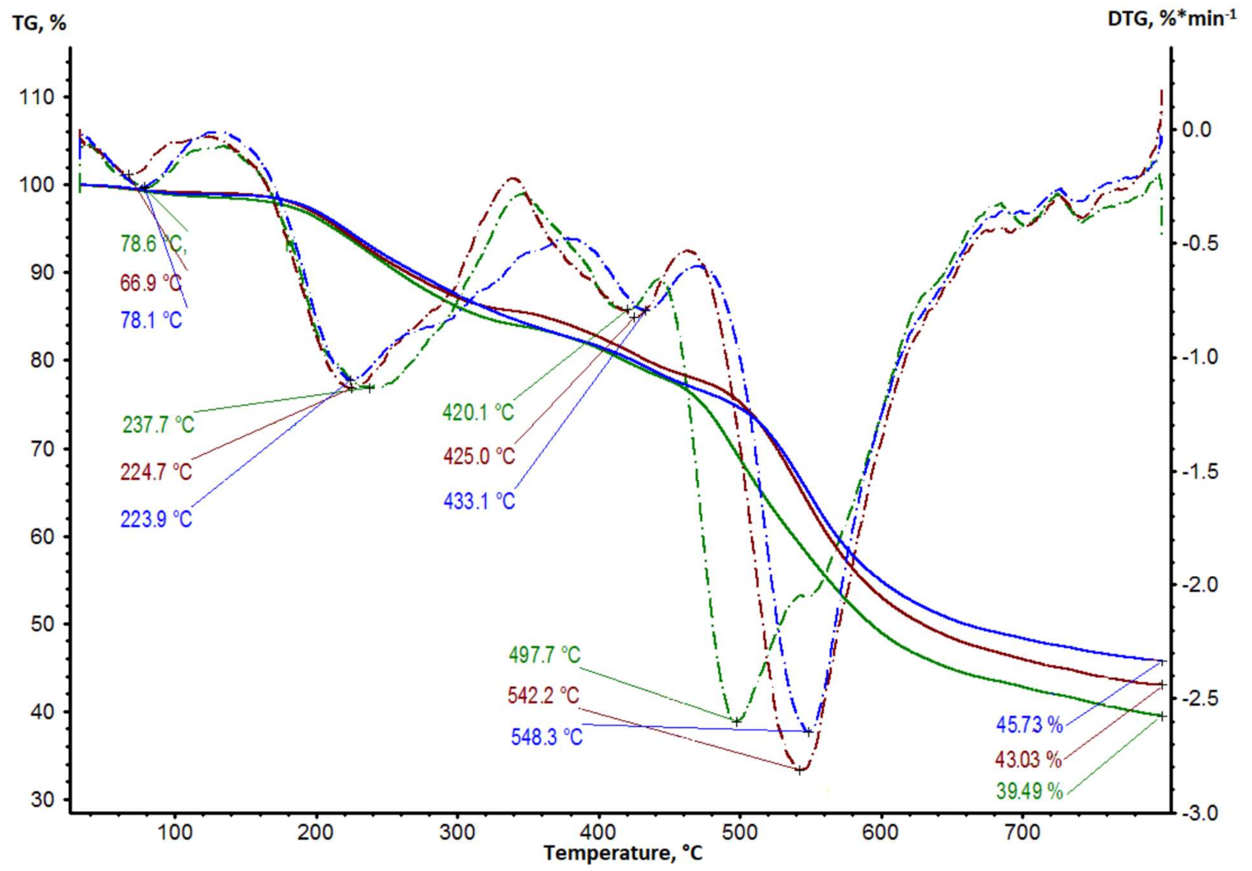

Figure S2. TG (solid lines) and DTG (dashed lines) curves of coPAI-1 (green), coPAI-2 (brown), coPAI-3 (blue).

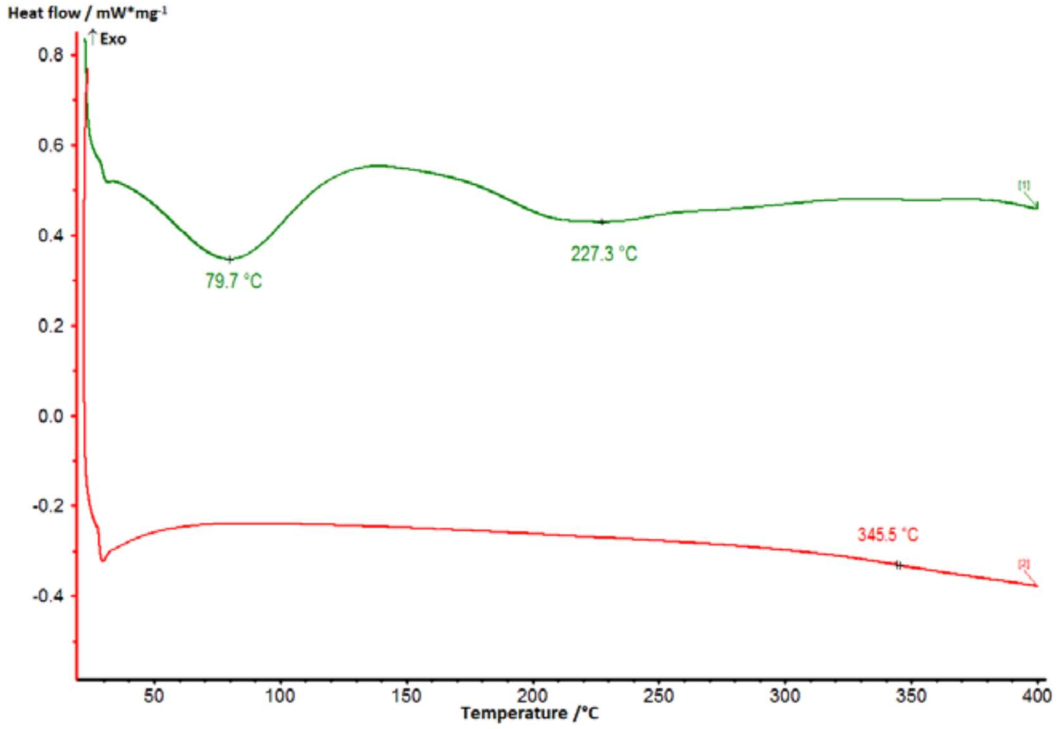

Figure S3a. 1-st and 2-nd DSC scan curves of coPAI-1.

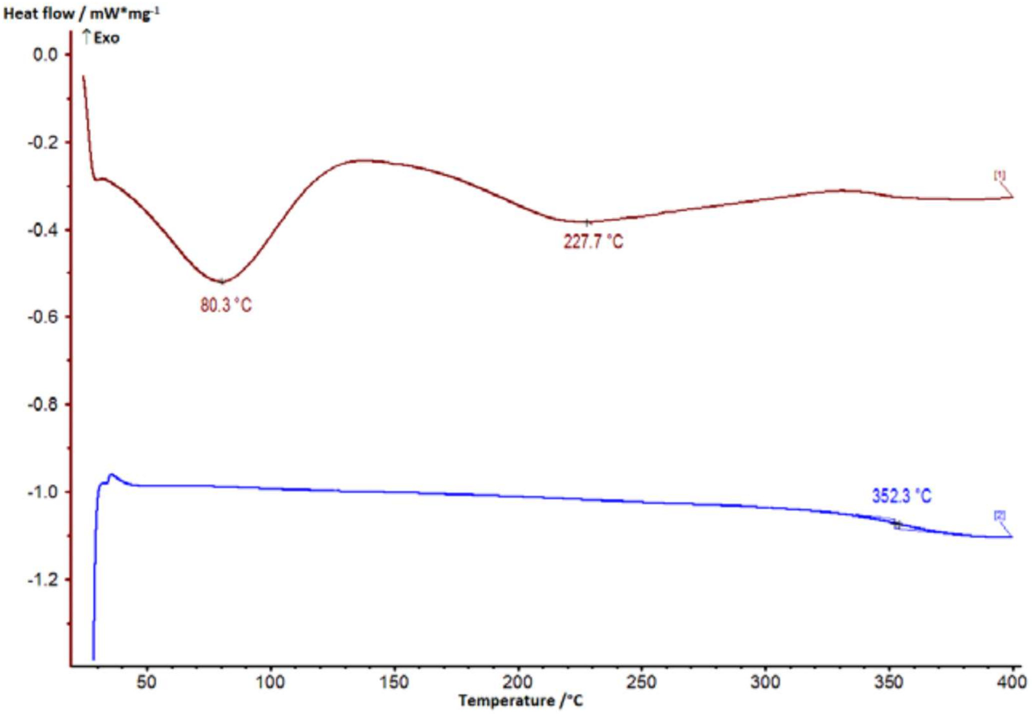

Figure S3b. 1-st and 2-nd DSC scan curves of coPAI-2.

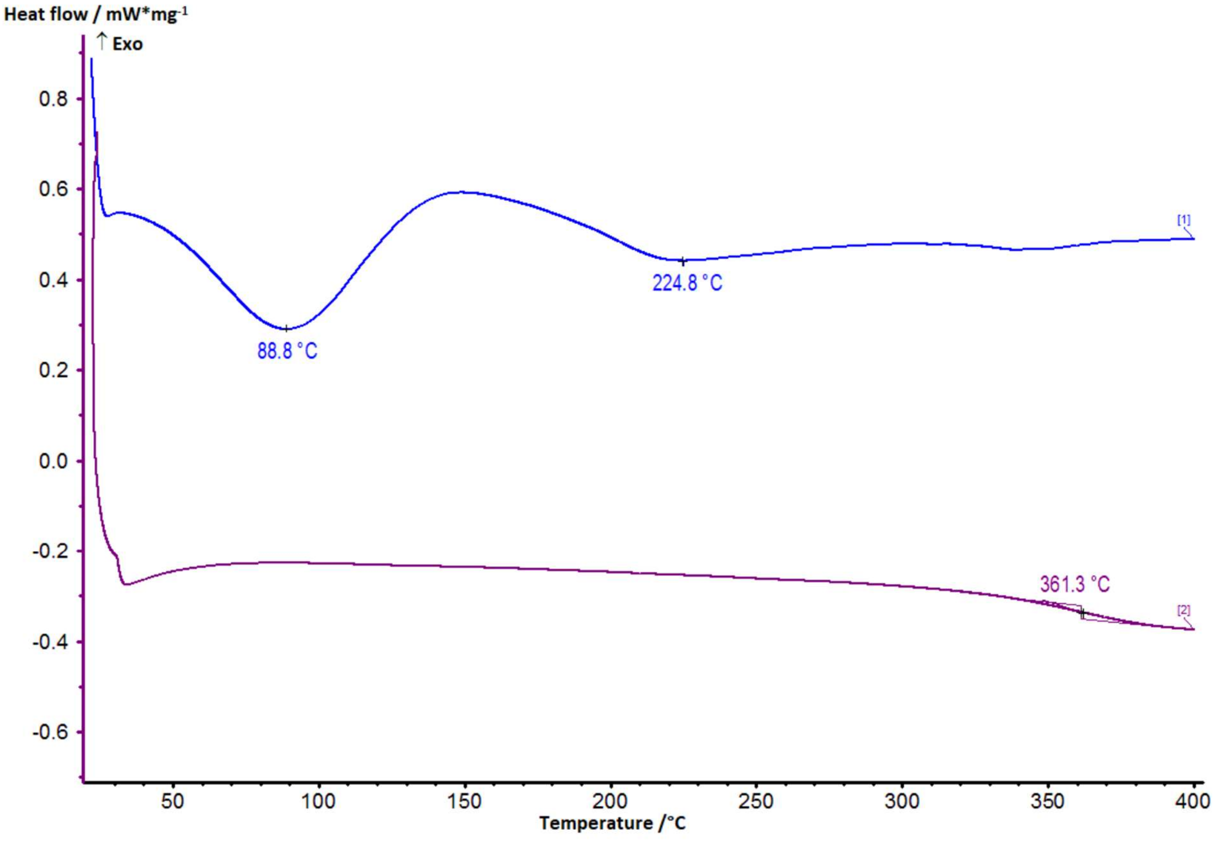

Figure S3c. 1-st and 2-nd DSC scan curves of coPAI-3.
